# Identification of amino acids restricting HBV receptor function in porcine NTCP

**DOI:** 10.1038/s44298-024-00041-5

**Published:** 2024-07-23

**Authors:** Samuel D. Jeske, Jochen M. Wettengel, Florian Gegenfurtner, Konrad Fischer, Judith Moosmüller, Anindita Chakraborty, Chunkyu Ko, Benjamin J. Burwitz, Angelika Schnieke, Ulrike Protzer

**Affiliations:** 1 Institute of Virology, School of Medicine and Health, Technical University of Munich/Helmholtz Munich, Munich, Germany; 2 German Center for Infection Research (DZIF), Munich partner site, Munich, Germany; 3 Division of Pathobiology and Immunology, Oregon National Primate Research Center, Oregon Health & Science University, Beaverton, OR USA; 4 Chair of Livestock Biotechnology, School of Life Sciences, Technical University of Munich, Freising, Germany

**Keywords:** Hepatitis B virus, Viral pathogenesis, Virus-host interactions

## Abstract

With 254 million chronically infected patients, hepatitis B virus (HBV) continues to be a severe health threat. While animal models play a crucial role in developing new therapies, the availability of preclinical HBV models is very limited. Therefore, novel in vivo infection models are urgently needed. The bona fide HBV receptor, sodium-taurocholate cotransporting polypeptide (NTCP), determines HBV’s species and cell-type specificity. Recent studies have indicated that the expression of human NTCP is the only limiting factor for HBV infection in selected species, such as macaques or pigs. Here, we confirm HBV infection of pig hepatocytes expressing human NTCP and show that porcine NTCP does not support HBV binding. By gradually humanizing porcine NTCP and site-directed mutagenesis, we identified amino acids 158 and 167 in porcine NTCP, limiting HBV interaction. In a proof-of-concept experiment, we showed that the expression of porcine NTCP with humanized amino acids 157-167 renders primary porcine hepatocytes fully susceptible to HBV. These results pave the way for generating transgenic pigs with humanized porcine chimeric NTCP as a novel, fully immunocompetent infection model for developing and validating new curative HBV therapies.

## Introduction

Hepatitis B virus (HBV) is an enveloped, partially double-stranded DNA virus belonging to the family of *Hepadnaviridae*. The WHO reports 254 million chronically infected patients worldwide, although an effective prophylactic vaccination is available^1^. These individuals are at high risk for developing progressive liver diseases such as liver fibrosis, cirrhosis, or hepatocellular carcinoma. Approximately 1.1 million deaths annually are attributed to HBV-related liver diseases. Despite significant achievements in HBV treatment, no reliable curative therapy for chronic HBV infection is available to date, and the development and evaluation of novel treatment options, especially targeting the virus’s persistence form, the nuclear covalently closed circular (ccc) DNA, are urgently needed^2,3^.

Availability of HBV animal models is highly limited since HBV naturally only infects humans and humanoid primates^4^. Chimpanzees were initially used as the only physiologically relevant, immunocompetent animal model, but a moratorium by international health agencies halted their use in research^5^. In 2012, Yan et al. identified NTCP as the bona fide receptor that enables binding of the PreS1 domain of the large surface protein and consecutively HBV cellular entry^6^. NTCP expression is the limiting factor for HBV susceptibility in human hepatoma cell lines such as HepG2 and Huh-7 and has also been shown to play a crucial role in HBV species specificity^7,8^. While naïve macaques are not susceptible to HBV, they support a complete HBV lifecycle following the hepatic expression of human NTCP (hNTCP) via adenoviral vectors^9^. In contrast, expression of hNTCP in mice leads to HBV binding and cellular uptake, but a yet unknown post-entry block prevents full permissivity for HBV infection^7,10^. Artificially introducing HBV into the mouse hepatocyte allows the establishment of a persistent HBV replication, but the natural transcription template is lacking, and the virus cannot spread^11^. Grafting mouse livers with human hepatocytes allows for HBV infection but requires the use of immunodeficient animals^12^. This limits their suitability since curative targeting of cccDNA persistence will most likely require activation of the HBV-specific immunity^2^. As neither cell culture nor immune-deficient animal models are sufficient to evaluate immune-based therapies, alternative models are urgently needed^4^.

Recently, a report from *Lempp* et al. showed that adeno-associated virus (AAV)-mediated expression of hNTCP in primary porcine hepatocytes (PPH) renders them fully permissive to HBV^13^. This qualifies pigs as a promising candidate species for establishing a new HBV animal model, preferably by modifying porcine NTCP (pNTCP) into a functional HBV receptor. Therefore, this study defines the amino acid (aa) exchanges required in pNTCP that allow pNTCP to fully function as an HBV receptor. We generated a chimeric porcine-human NTCP (phNTCP) with humanized aa 157-168 and showed that its expression allows human hepatoma cell line HepG2 and porcine hepatocytes to support HBV infection and initiate a complete HBV life cycle. These results provide the basis for a targeted alteration of pNTCP in order to transform pigs into a novel, immunocompetent animal model supporting HBV infection.

## Results

### Expression of hNTCP renders primary pig hepatocytes susceptible to HBV infection

**Fig. 1:**
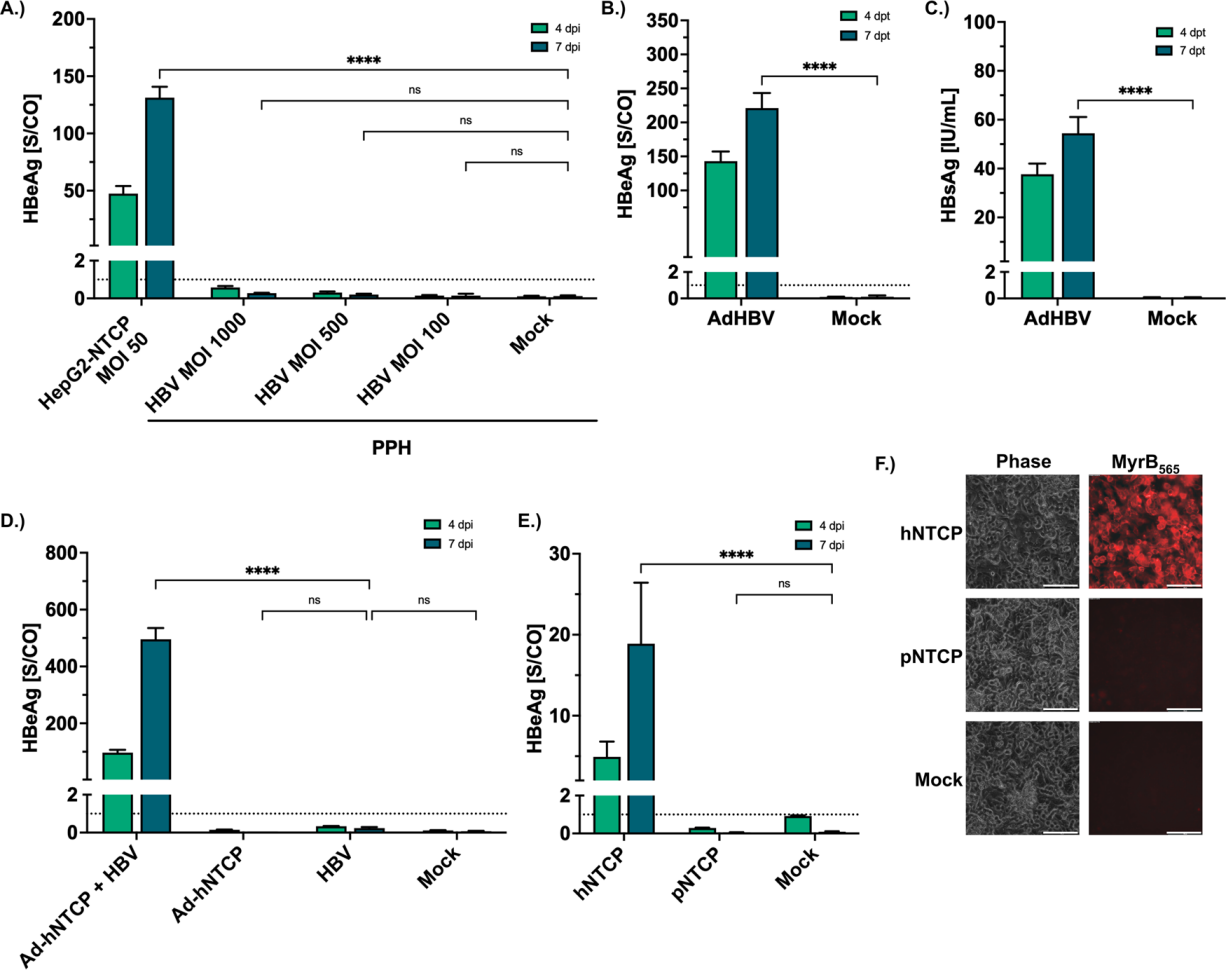
Expression of hNTCP is the limiting factor for HBV infection in PPH. **A**–**D** PPH were isolated from fresh pig liver using the two-step collagenase perfusion method, seeded into 12-well plates, and differentiated with 2% DMSO for 48 h. (**A**) Differentiated PPH were inoculated with HBV at indicated MOI. PPH not inoculated with HBV served as negative (Mock) and HepG2-NTCP (K7) cells inoculated with HBV (MOI 50 vp/cell) as positive controls. Cell culture medium was collected at days 4 and 7 post-inoculation (dpi) and analyzed for HBeAg. **B**, **C** Differentiated PPH were transduced via an adenoviral vector carrying a 1.3-fold HBV genome. Cell culture medium was collected at days 4 and 7 post-transduction (dpt) and analyzed for HBeAg (**B**) and HBsAg (**C**). **D** PPH were transduced with an adenoviral vector expressing hNTCP and subsequently inoculated with HBV at MOI 1000 vp/ml. Cell culture medium was collected at days 4 and 7 post-infection and analyzed for HBeAg. **E**, **F** HepG2 cells were transfected with mRNA encoding hNTCP or pNTCP and differentiated with 2% DMSO for 48 h. **E** Cells were inoculated with HBV (MOI 300 vp/cell), and cell culture medium was collected and analyzed for HBeAg at days 4 and 7 post-infection. **F** Cells were treated with fluorescently labeled Myrcludex B (MyrB_565_) and analyzed via bright field (phase contrast) and fluorescence microscopy (scale bar: 100 µm). Representative images are shown. **A–E** Dotted lines indicate the cut-off between non-reactive and reactive. Experiments were performed in biological triplicates; mean values +/− standard deviation are given. Statistical analysis was performed by one-way ANOVA with Dunnett’s multiple comparison correction (**A**, **D**, **E**) or *t*-test (**B**, **C**). ****p < 0,0001, ns = not significant.

We isolated PPH from fresh pigs’ livers to evaluate their HBV permissivity. We confirmed adherence and cell morphology on day 1 after isolation (Supplementary Fig. 1). We inoculated the cells with increasing HBV multiplicities of infection (MOI) and analyzed cell culture supernatants for HBeAg on day 4 and day 7 post-inoculation (Fig. 1A), but no HBeAg was detectable. This indicates that naive PPH are not permissive for HBV. Next, we investigated the capability of PPH to support HBV gene expression after transduction with an adenoviral vector encoding a replication-competent 1.3-fold HBV genome (Ad-HBV) together with a GFP expression cassette. GFP expression confirmed the successful transduction of the PPH (Supplementary Fig. 2). Following Ad-HBV infection, we detected high levels of HBeAg and HBsAg in the supernatant (Fig. 1B, C), indicating that PPH support HBV RNA transcription and gene expression from the nuclear HBV template.

To narrow down the reason for the blocking of HBV entry, we used an adenovirus expressing the HBV entry receptor hNTCP (Ad-hNTCP) to transduce PPH. After Ad-hNTCP transduction, cells were inoculated with HBV at a high MOI of 1000 viral particles per cell (vp/cell), and cell culture supernatants were analyzed for HBeAg to confirm productive infection. We detected high levels of HBeAg in the supernatant of HBV-infected PPH expressing hNTCP (Fig. 1D), indicating that the limiting factor for a productive HBV infection had been overcome.

To compare hNTCP and its porcine ortholog, we isolated the total RNA from PPH (*sus scrofa*), generated cDNA, and cloned the porcine NTCP (pNTCP) coding sequence into an expression plasmid. Sequence comparison of hNTCP and pNTCP showed multiple differences in nucleotide (Supplementary Table 1) and aa sequences (Supplementary Table 2). We next generated mRNA of both pNTCP and hNTCP transfected HepG2 cells and inoculated the transfected cells with HBV. As expected, only the cells expressing hNTCP but not the cells expressing pNTCP secreted HBeAg (Fig. 1E), indicating that pNTCP does not support HBV infection.

To determine whether HBV does not bind to pNTCP or viral entry is impaired at a post-binding step, we stained the transfected cells with the fluorescently labeled PreS1-derived peptide Myrcludex B (MyrB_565_). The lack of MyrB_565_ fluorescence signal indicates that HBV does not bind to pNTCP (Fig. 1F).

### Amino acid exchanges S158G and P167L in pNTCP enable HBV binding and infection

**Fig. 2:**
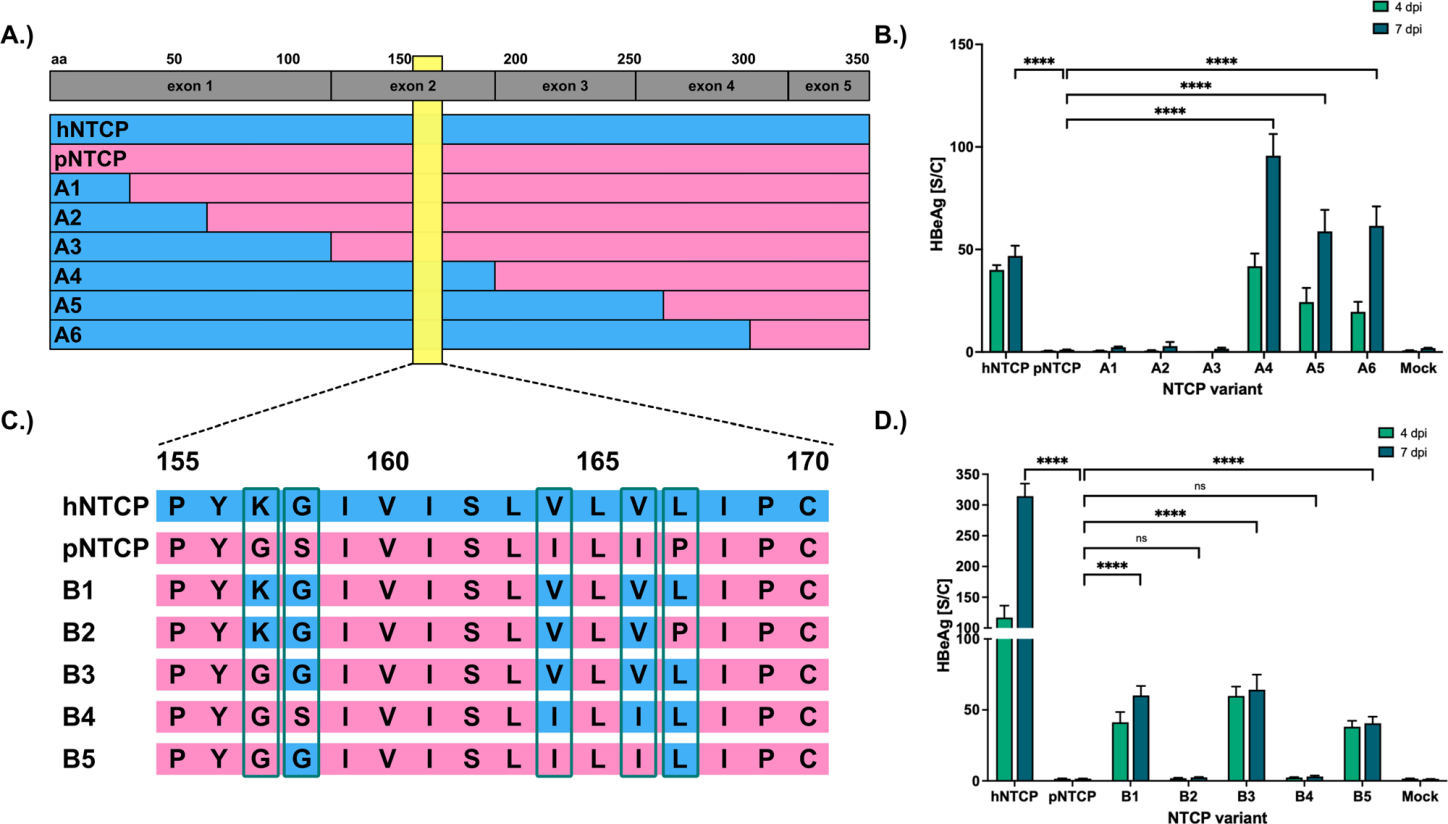
Identification of the amino acids to be exchanged in pNTCP for HBV infection. **A** Overview of the NTCP aa sequences encoded by the generated mRNA (A1–A6) with a gradual exchange of larger regions of the pNTCP sequence with their human counterparts. **B** HepG2 cells were transfected with the generated mRNAs (A1–A6), differentiated with 2% DMSO for 48 h, and subsequently inoculated with HBV (MOI 500 vp/cell). Cell culture supernatants were collected at days 4 and 7 post-infection and analyzed for HBeAg. **C** Overview of the NTCP aa sequences encoded by the generated mRNA (B1-B5) based on pNTCP with aa exchanges in the aa region 155–170. **D** HepG2 cells were transfected with the generated mRNAs (B1-B5), differentiated with 2% DMSO for 48 h, and subsequently inoculated with HBV (MOI 500 vp/cell). Cell culture supernatants were collected at days 4 and 7 post-infection and analyzed for HBeAg. **B**, **D** Experiments were performed in biological triplicates; mean values +/− standard deviation are given. Data were analyzed by one-way ANOVA with Dunnett’s multiple comparison correction. ****p < 0,0001, ns = not significant. B1 = phNTCP (G157K; S158G; I164V; I166V; P167L); B2 = phNTCP (G157K; S158G; I164V; I166V); B3 = phNTCP (S158G; I164V; I166V; P167L); B4 = phNTCP (I164V; I166V; P167L); B5 = phNTCP (S158G; P167L).

We next examined the aa differences between the hNTCP and pNTCP orthologs to identify the functional domains of pNTCP restricting HBV binding and infection. Therefore, we created various chimeric phNTCP constructs by gradually replacing the aa sequences of pNTCP with their human counterparts (Fig. 2A). HepG2 cells were then transfected with mRNA encoding for these constructs and stained with MyrB_565_ (Supplementary Fig. 3A). Notably, this staining revealed that phNTCP variants containing a humanized exon 2 allow for the binding of MyrB_565_. Next, we repeated the transfection of HepG2 cells, inoculated the cells with HBV, and analyzed the supernatants for HBeAg. In concordance with the previous results, HBeAg could be detected in the supernatant of cells transfected with phNTCP variants with a humanized exon 2 (Fig. 2B), indicating that a crucial domain for HBV binding to NTCP and viral entry is located in this exon. We confirmed our finding by systematically exchanging aa sequences of hNTCP with their porcine counterparts (Supplementary Fig. 4). As expected, hNTCP with a porcine exon 2 sequence did not support HBV infection.

By comparing the exon 2 aa sequences between hNTCP and pNTCP, we found that the orthologs deviate in three aa (K157G, G158S, V164I) in the previously described region aa 157-165^6^ (Supplementary Table 2), and in two additional aa, V166I and L167P, in close proximity to this region. To determine the role of these five aa alterations, we created further phNTCP constructs by substituting single aa of pNTCP with their human counterparts (Fig. 2C). HepG2 cells were then transfected with mRNA encoding for the chimeric phNTCP constructs, inoculated with HBV, and the supernatants were analyzed for HBeAg (Fig. 2D). We found HBeAg in the supernatants of cells expressing phNTCP with humanized aa 157-167 and could narrow down the block to the two aa, S158G and P167L. These results were confirmed by MyrB_565_ binding to the transfected cells (Supplementary Fig. 3B) and is in concordance with the HBeAg secretion results. From this, we concluded that humanizing aa 157-167, especially S158G and P167L, renders pNTCP fully functional for HBV binding and infection.

### Generation of stable cell lines expressing NTCP variants

**Fig. 3:**
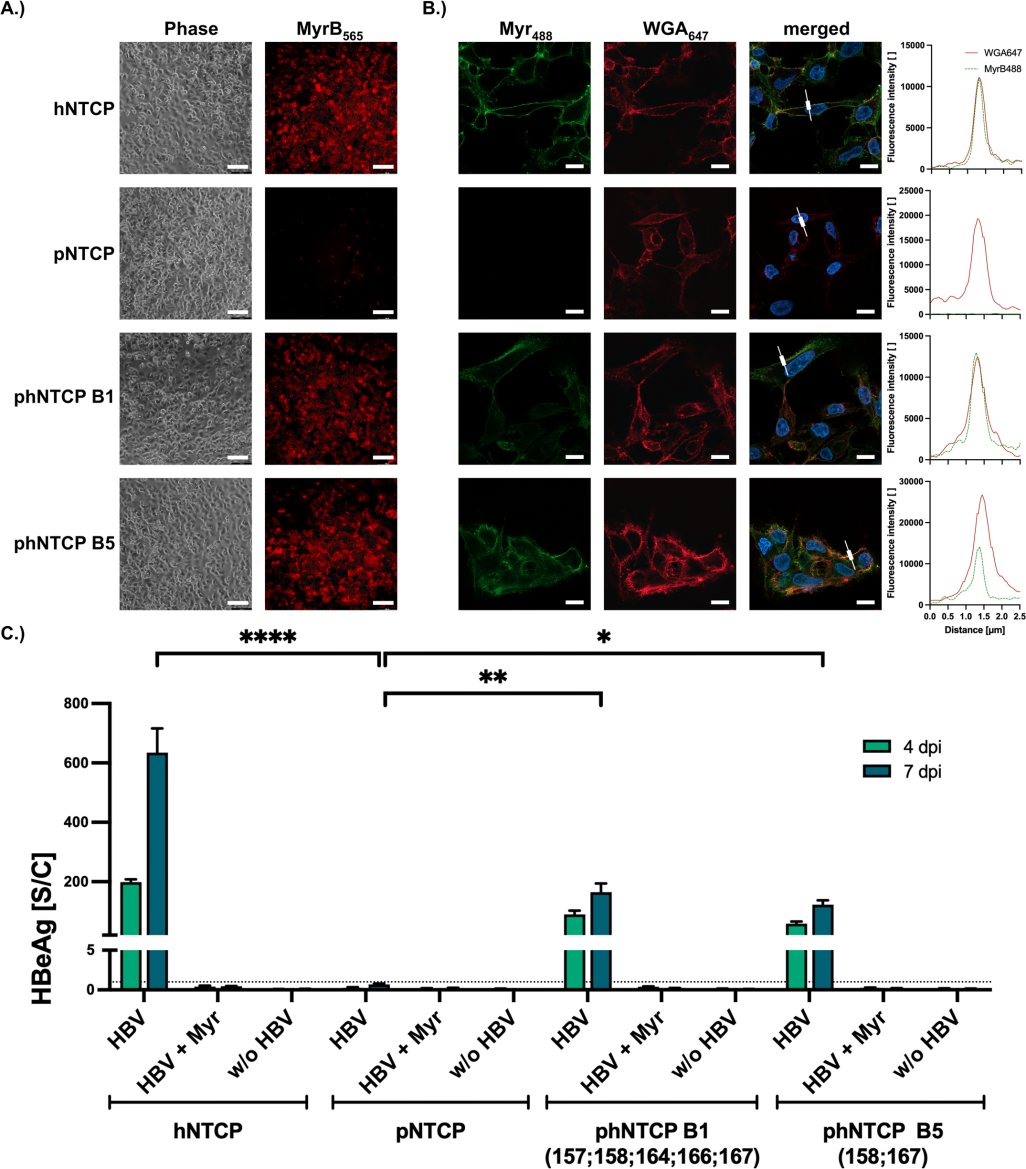
Generation of stable cell lines expressing hNTCP, pNTCP, and phNTCP variants (157-167) and (S158G; P167L). HepG2 cell lines stably expressing hNTCP, pNTCP, phNTCP B1 (157-167), and phNTCP B5 (S158G; P167L) were generated. **A** Cells were treated with fluorescently labeled MyrB_565_ and analyzed via fluorescence microscopy to investigate HBV binding (scale bar: 100 µm). **B** Cells were seeded with low density and treated with Atto_488_-labeled MyrB (MyrB_488_). Membrane structure was visualized with Alexa Fluor_647_ labeled wheat germ agglutinin (WGA_647_), and nuclei were stained with Hoechst33342. High-resolution confocal microscopy was performed to investigate the correct location of the NTCP variants expressed (scale bar: 10 µm). In order to visualize the colocalization of WGA_647_ and MyrB_488_, fluorescence intensities along white indicators were compared. **C** Cells were seeded, differentiated, and inoculated with HBV (MOI 500 vp/cell). Supernatants were collected and analyzed for HBeAg at days 4 and 7 post-infection. The dotted line indicates the cut-off between non-reactive and reactive. Experiments were performed in biological triplicates; mean values +/− standard deviation are given. Data were analyzed by one-way ANOVA with Dunnett’s multiple comparison correction. *p < 0.05, **p < 0.01, ****p < 0.0001.

To compare the HBV receptor functions of hNTCP, phNTCP (157-167), and phNTCP (S158G; P167L) independently of the number of transfected cells, we generated HepG2-based cell lines stably expressing the different NTCP variants. We first analyzed MyrB_565_ binding and could confirm its binding to hNTCP and both chimeric phNTCP variants, phNTCP (157-167) and phNTCP (S158G; P167L), but not to pNTCP (Fig. 3A). Using confocal microscopy, we detected a membrane-bound localization of MyrB_565_ in hNTCP and both phNTCP versions indicating correct folding and surface transport of the chimeric proteins (Fig. 3B).

We then inoculated the cell lines with HBV and analyzed cell culture supernatants for HBeAg. Cells stably expressing hNTCP and both phNTCP (157-167) and phNTCP (S158G; P167L) variants could be infected and secreted detectable levels of HBeAg in contrast to the cells expressing non-modified pNTCP. Pre-treatment with MyrB prevented HBV infection (Fig. 3C). These results confirm that the two chimeric phNTCP variants support HBV infection, however, to a lesser extent than hNTCP.

**Fig. 4:**
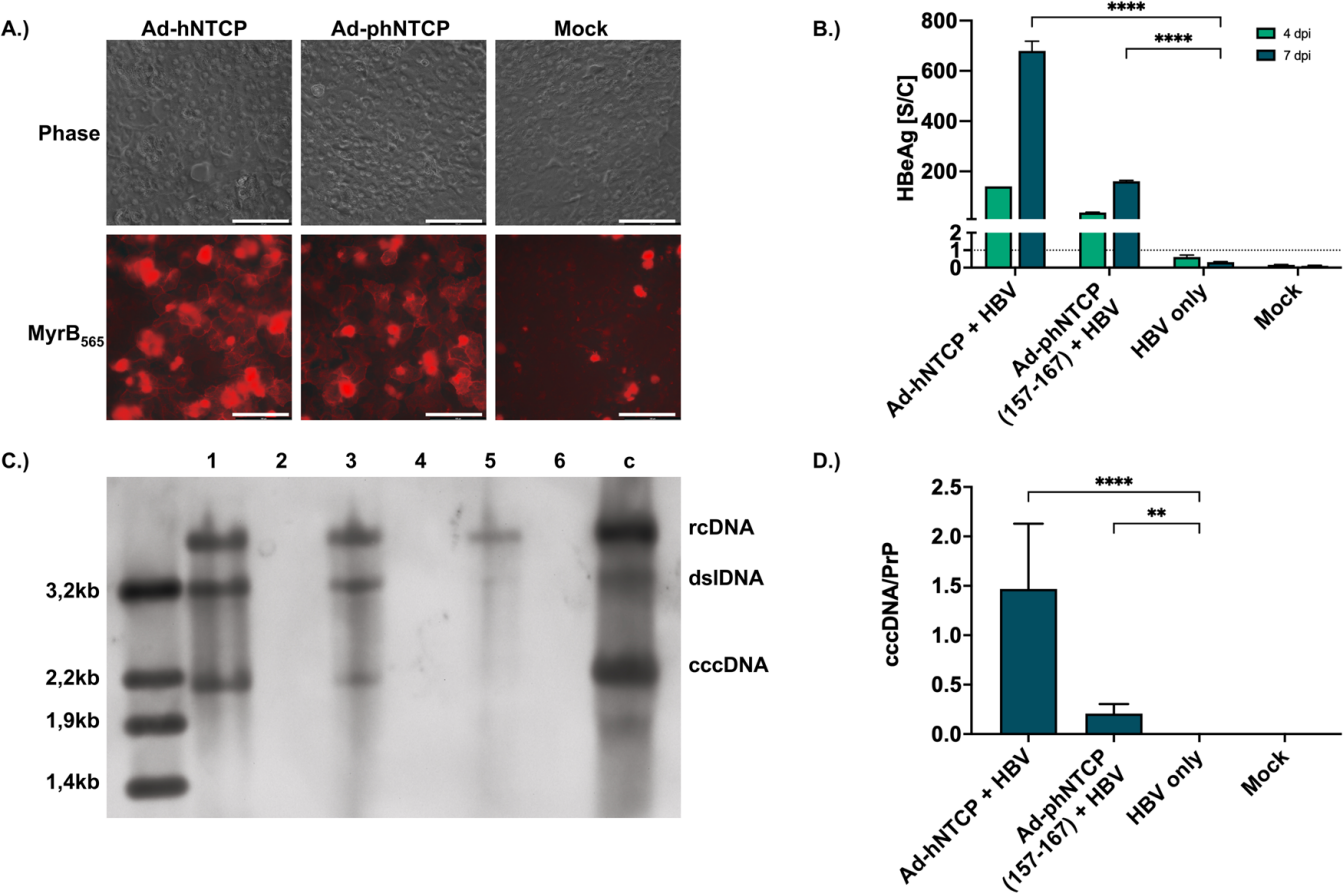
HBV infection of primary porcine hepatocytes with partially humanized porcine NTCP. PPH were isolated using a 2-step collagenase method and seeded on a 24-well plate. Cells were allowed to attach for 24 h, transduced with adenoviral vectors expressing hNTCP or phNTCP (157-167), and subsequently differentiated in DMSO 2% for 48 h. **A** Cells were stained with fluorescently labeled Myrcludex B (Myr_565_) and analyzed via fluorescence microscopy, showing binding to the NTCP variants expressed on the cell surface (scale bars: 100 µm). **B**–**D** In parallel, cells were inoculated with HBV (MOI 1000 vp/cell). **B** Cell culture supernatants were collected at days 4 and 7 post-infection and analyzed for HBeAg. **C** 7 days post-infection, southern blot analysis was performed utilizing a modified Hirt extraction procedure. Analyzed samples include (1) PPH transduced with Ad-hNTCP and inoculated with HBV, (2) PPH transduced with Ad-hNTCP only, (3) PPH transduced with Ad-phNTCP (157-167) and inoculated with HBV, (4) PPH transduced with Ad-phNTCP (157-167) only, (5) naive PPH inoculated with HBV, (6) naive PPH without HBV, (c) HBV infected HepG2-NTCP as a positive control. **D** 7 days post-infection, cells were lysed, total DNA was isolated from the lysate and analyzed for cccDNA via PCR. Values are displayed in relation to prion protein gene. **B**, **D** Mean values +/− standard deviation of at least three experiments are given. Statistical analysis was performed by (**B**) one-way ANOVA with Dunnett’s multiple comparison correction or (**D**) Kruskal–Wallis test with Dunn’s multiple comparison correction. **p < 0,01, ****p < 0,0001. For (**B**), the dotted line indicates the cut-off between non-reactive and reactive.

Since both chimeric phNTCP variants support HBV infection with lower efficacy than hNTCP, we aimed to show whether the expression of the chimeric phNTCP variants is sufficient to render PPH susceptible to HBV. As the phNTCP (157-167) variant showed a higher efficacy in the previous experiment (Fig. 3C), we created an adenoviral vector encoding for phNTCP (157-167) (Ad-phNTCP (157-167)) and transduced PPH. PPH expressing phNTCP (157-167) bound MyrB_565_ (Fig. 4A) and secreted HBeAg after HBV inoculation (Fig. 4B), indicating that the expression of the chimeric phNTCP (157-167) is sufficient to allow HBV infection of PPH.

To confirm this HBV infection, PPH were lysed and analyzed for the presence of cccDNA via Southern blot. Southern blot analysis proved the presence of cccDNA in PPH expressing hNTCP or the chimeric phNTCP (157-167) (Fig. 4C). Notably, relaxed circular DNA (rcDNA) and double-stranded linear DNA (dslDNA), but not cccDNA, were also found in non-transduced cells inoculated with HBV, most likely originating from the input virus. The presence of cccDNA could be confirmed by a cccDNA-selective qPCR in both hNTCP and phNTCP (157-167) expressing PPH upon HBV infection, however, with a significantly lower amount in the phNTCP (157-167) expressing cells (Fig. 4D). In addition, we quantified rcDNA levels in the cell culture supernatant of the infected PPH and detected 1 × 10^7^ HBV genome equivalents/ml (Supplementary Fig. 5). This was comparable to the amount detected after infection of hNTCP expressing primary macaque hepatocytes^9^, but >10-fold lower than the amount released from HBV-producing cell lines^14^. When inoculating PPH transduced with hNTCP and phNTCP (157-167) with the cell culture supernatant from 4 dpi, we were neither at 4 nor 7 dpi able to prove transmission of HBV infection. This was most likely due to the low MOI of only 10 genome equivalents/ml that could be used in this setting.

Taken together, our results confirmed the establishment of HBV cccDNA in HBV-inoculated PPH expressing hNTCP or phNTCP (157-167), demonstrating that PPH can support a full HBV replication cycle after the expression of either hNTCP or chimeric phNTCP (157-167).

## Discussion

The urgent need for novel animal models in HBV research remains, and their development is the subject of extensive investigations. In this study, we verified that the overexpression of hNTCP renders PPH fully permissive to HBV. Using mRNA transfection and adenoviral vector transduction, we proved that pNTCP does not confer HBV permissivity because it does not allow for HBV binding. We found that exchanging aa 157-167 in pNTCP with their human counterparts, particularly substituting aa S158G and P167L, yielded a chimeric phNTCP that permits both HBV binding and infection.

It has previously been reported that overexpression of hNTCP in macaque and pig hepatocytes allows for infection with HBV and hepatitis delta virus (HDV)^13^. This initiated considerations about using macaques and pigs as novel immune-competent animal models for HBV infection. While the generation of transgenic macaques expressing hNTCP is currently being established, pigs could serve as a promising HBV animal model due to their anatomical and physiological similarity to humans, their broad use in livestock, and elaborated techniques to genetically modify these animals^15^.

An ideal porcine HBV animal model depends on the germline expression of a functional NTCP supporting HBV binding and uptake. However, overexpression of full hNTCP may lead to metabolic changes or cellular toxicity in transgenic animals if NTCP is expressed outside of hepatocytes or if the corresponding protein transporting bile acids out from hepatocytes into the bile is not expressed at equal levels. Therefore, the preferable approach is the precise alteration of pNTCP into a functional HBV receptor that maintains the physiological regulation of the bile acid transporters. Since a comprehensive understanding of the aa blocking HBV binding and infection in pNTCP is crucial for utilizing pigs as HBV model animals, we examined the sequence differences between the human and porcine NTCP orthologs in detail.

Our results confirm the previous findings that the aa region 157-165 is critical for HBV binding and infection^6^, but underline the importance of other nearby aa to form an extracellular region of NTCP capable of binding HBV^16,17^. Notably, another extracellular region located around 30 Å distance^18^, formed by aa 84-87, has been identified in murine NTCP and is crucial for HBV infection^10^. This region in the TM2-TM3 loop has been recently shown to bind the C-terminal PreS1 domain^17^. Although pNTCP also differs from hNTCP in this region in aa K86N, our results indicate that this alteration does not impede HBV infection. However, a direct comparison of HBeAg levels after HBV infection of hepatoma cells expressing our chimeric NTCP variants, phNTCP (157-167) and phNTCP (S158G; P167L), revealed an inferiority of the chimeric phNTCP variants compared to hNTCP in terms of HBV infection. The presumed influence of aa K86N on HBV-NTCP interaction also concurs with previous studies, which showed that implementing this mutation and others in hNTCP reduces the rate of viral infection in cell culture^19^. Although further post-entry impairments in combination with a reduced entry may prevent HBV infection, our results prove that the expression of the chimeric phNTCP (157-167) in PPH is sufficient to establish a full HBV replication cycle, including cccDNA formation.

This report highlights the possibility of exchanging aa 157-167 in pNTCP in vivo to generate a transgenic pig using novel technologies such as CRISPR-mediated homology-directed repair. It paves the way for the generation of a transgenic pig that can serve as a fully immunocompetent HBV infection model.

## Methods

### Genetic background of pigs

For this study, German Landrace pigs (*sus scrofa*) and hybrid pigs, generated by crossbreeding German Landrace pigs and Black Forest minipigs, were used. Pigs were euthanized for other experimental settings and tissue sample isolation.

### RNA isolation and cDNA synthesis

RNA was isolated based on the High Pure RNA Isolation Kit (Roche, Germany) and cDNA was synthesized using the SuperScript II kit (Thermo Fisher Scientific, Germany) according to the manufacturer’s instructions. GoTaq DNA polymerase kit (Promega, Germany) was used for the amplification of NTCP-specific regions.

### Sequencing

Sequencing was performed by MWG Eurofins, Germany using NTCP-specific primer combinations.

### PPH isolation

A piece of pigs’ liver harvested from freshly sacrificed animals was flushed with 500 ml of HBSS containing EGTA and subsequently continuously perfused with 250 ml DMEM/F12 containing 50 µg/ml Collagenase NB8 for 1 h at 37 °C. To collect the hepatocytes, the liver capsule was dissected with a scalpel, and the organ was gently rinsed with cell culture medium to create a cell suspension. This suspension was filtered firstly through a tea strainer to remove larger fragments and secondly through a 100 µm nylon cell strainer to establish a single-cell suspension. Cells were washed three times by centrifugation at 100 × *g* and resuspended with cell culture medium at 4 °C. To remove non-vital hepatocytes, a 40% Percoll gradient was utilized to purify the cell suspension. The vital hepatocytes were resuspended in cell culture medium containing 10% FBS at 37 °C, counted, and plated onto collagenized 24-well plates at a density of 5 × 10^5^ cells per well. Cells were allowed to attach overnight, washed three times with PBS, and subsequently cultured in an FCS-free maintenance medium.

### Production of HBV and infection of cells

HBV stocks were purified from the cell culture supernatant of stable HBV-producer cell lines HepAD38 by heparin affinity chromatography followed by sucrose gradient ultracentrifugation according to published protocols^14^. If not otherwise stated, cells and cell lines were differentiated using 2% DMSO for 48 h and subsequently infected with HBV (MOI 200 vp/cell) in the presence of 4% polyethylene glycol (PEG) 8000 for 24 h. To analyze HBV spread, cell culture supernatants were diluted 1:1 with 8% PEG 8000. After infection, cells were washed and cultured with 2% DMSO. To block HBV infection, MyrB was added at a concentration of 200 nM to selected experiments. Cells were MyrB pretreated for 2 h and for the time of HBV inoculation.

### Generation and use of recombinant adenoviral vectors

Adenoviral vectors for the expression of different NTCP variants were generated using the pAD/PL-DEST Gateway vector system (Thermo Fisher Scientific), based on the E1- and E3-deleted human adenovirus serotype 5. For this, NTCP sequences were cloned in a hepatic expression cassette (TTR promoter – NTCP variant – BGH poly A) on a pENTER vector^20^. Adenoviral stocks were generated by transfecting the PacI-digested adenoviral plasmids on Hek293 cells using Lipofectamine 3000 (Thermo Fisher Scientific) and further amplified as previously described. If not otherwise indicated, cells were transduced with the adenovirus vector by adding a stock volume equivalent to the specific MOI for 24 h.

### mRNA synthesis and transfection

mRNA was generated using the HiScribe T7 ARCA mRNA Kit with tailing (New England Biolabs, Germany) according to the manufacturer’s instructions and adapted as previously described^21^. Transfection of cells with the generated mRNA was performed using the Lipofectamine MessengerMAX (Thermo Fischer Scientific) transfection reagent according to the manufacturer’s instructions.

### HBV serology

HBeAg was measured using an automated BEP III system (Siemens Healthcare, Germany). The results of HBeAg analyses are presented as sample/cut-off (S/CO) values using the internal cut-off value of the measurement. Quantification of HBsAg was performed using the Abbott Architect platform (Abbott Diagnostics, Ireland).

### PCR

Cellular DNA was extracted using the NucleoSpin Tissue kit (Macherey-Nagel, Germany). For cccDNA detection, isolated DNA was treated with 5 units of T5 exonuclease (New England Biolabs) for 30 min, followed by heat-inactivation at 95 °C for 5 min. PCR amplification was performed on a LightCycler480 instrument (Roche) using forward primer (5′-GCCTATTGATTGGAAAGTATGT-3′) and reverse primer (5′-AGCTGAGGCGGTATCTA-3′). For quantification, an external plasmid standard was used. For relative quantification, the human prion protein (PRNP) gene was amplified on a LightCycler480 instrument (Roche) using forward primer (5′-TGCTGGGAAGTGCCATGAG-3′) and reverse primer (5′-CGGTGCATGTTTTCACGATAGTA-3′).

### Southern blot analysis

HBV cccDNA was detected using a modified Hirt extraction procedure followed by a Southern blot analysis as previously described^11^. In short, HBV DNA was separated on an agarose gel, transferred onto a nylon membrane, and hybridized with a digoxigenin-labeled HBV-specific probe. The probe signal was detected using a DIG Luminescent Detection Kit.

### Analysis of NTCP using fluorescently labeled Myrcludex B

PreS1 binding was visualized using Atto_647_, Atto_565_ or Atto_488_ labeled Myrcludex B as previously described^6^.

### Generation of NTCP expressing stable cell lines

For the generation of stable HepG2 cell lines expressing different NTCP variants, the PiggyBac technology, in combination with an antibiotic selection procedure, was used as previously described^22^. In short, HepG2 cells were transfected with a plasmid encoding the PiggyBac transposase and a plasmid encoding the TTR-NTCP-BGH expression cassette flanked by PiggyBac-ITRs in the ratio 1:3. Stably transfected cell lines were selected using puromycin (10 mg/ml) (dilution 1:2000).

## Supplementary information

Supplementary material

## Data Availability

All relevant data are included in this manuscript and its supplementary information files. Additional data are available from the corresponding author (S.D.J.) upon request.
