# Supplementary material for: Identification of amino acids restricting HBV receptor function in porcine NTCP
Source: Npj Viruses. 2024 Jul 23;2:30. doi: 10.1038/s44298-024-00041-5 (PMC11721346; doi:10.1038/s44298-024-00041-5)
Supplement: Supplementary file 1 — Supplementary material [file 44298_2024_41_MOESM1_ESM.pdf]

Supplementary material

Supplementary Table 1: cDNA alignment of hNTCP and pNTCP

```
CLUSTAL O(1.2.4) multiple sequence alignment


hNTCP           ATGGAGGCCCACAACGCGTCTGCCCCATTCAACTTCACCCTGCCACCCAACTTTGGCAAG      60
pNTCP           ATGGAGGCCCTCAACGAGTCCGCTCCCATCAACTTCACCCTCCCGCACAACTTCGGCAAA      60
                ********** ***** *** ** **  ************* ** * ****** *****

hNTCP           CGCCCCACAGACCTGGCACTGAGCGTCATCCTGGTGTTCATGTTGTTCTTCATCATGCTC      120
pNTCP           CGGCCCACAGACCTGGCTCTGAGCGTCATCCTGGTGTTCATGCTGCTCATCATCATGCTC      120
                ** ************** ************************ ** ** ***********

hNTCP           TCGCTGGGCTGCACCATGGAGTTCAGCAAGATCAAGGCTCACTTATGGAAGCCTAAAGGG      180
pNTCP           TCCCTGGGCTGCACCATGGAGTTCGGCAGGATCAGGGCGCACTTTCGGAAACCTAAGGGA      180
                ** ********************* *** ***** *** *****  **** ***** **

hNTCP           CTGGCCATCGCCCTGGTGGCACAGTATGGCATCATGCCCCTCACGGCCTTTGTGCTGGGC      240
pNTCP           CTGGCCATCGCCCTGGTGGCGCAGTACGGCATCATGCCCCTCACTGCCTTTGCACTGGGC      240
                ******************** ***** ***************** *******  ******

hNTCP           AAGGTCTTCCGGCTGAAGAACATTGAGGCACTGGCCATCTTGGTCTGTGGCTGCTCACCT      300
pNTCP           AAGCTCTTCCGACTGAACAATGTCGAGGCGCTGGCCATCCTGATCTGTGGCTGCTCACCT      300
                *** ******* ***** **  * ***** ********* ** *****************

hNTCP           GGAGGGAACCTGTCCAATGTCTTCAGTCTGGCCATGAAGGGGGACATGAACCTCAGCATT      360
pNTCP           GGGGGGAACCTCTCCAACATCTTCGCTCTGGCCATGAAGGGGGACATGAACCTCAGCATC      360
                ** ******** *****  *****  *********************************

hNTCP           GTGATGACCACCTGCTCCACCTTCTGTGCCCTTGGCATGATGCCTCTCCTCCTGTACATC      420
pNTCP           ATGATGACCACCTGCTCCACCTTCCTTGCCCTGGGCATGATGCCCCTCCTCCTATACCTT      420
                 ***********************  ****** *********** ******** *** *

hNTCP           TACTCCAGGGGGATCTATGATGGGGACCTGAAGGACAAGGTGCCCTATaaaggcatcgtg      480
pNTCP           TACTCCAGGGGCATCTATGATGGGACCCTGAAGGACAAGGTGCCCTATGGCAGCATTGTG      480
                *********** ************  **********************    **** ***

hNTCP           atatcactggtcctgGTTCTCATTCCTTGCACCATAGGGATCGTCCTCAAATCCAAACGG      540
pNTCP           ATATCACTGATCCTGATTCCCATTCCCTGCACCATAGGCATCATCCTCAACACTAAACGG      540
                ********* ***** *** ****** *********** *** *******  * ******

hNTCP           CCACAATACATGCGCTATGTCATCAAGGGAGGGATGATCATCATTCTCTTGTGCAGTGTG      600
pNTCP           CCACAATATGTGCGCTATGTCATCAAGGGAGGAACGATCCTCTTGATTTTGTGCGCTATT      600
                ********  ********************** * **** ** *  * ******  * *

hNTCP           GCCGTCACAGTTCTCTCTGCCATCAATGTGGGGAAGAGCATCATGTTTGCCATGACACCA      660
pNTCP           GCTGTCACAGTGCTCTCTGTCCTCAACGTGGGCAAGAGCATCTTGTTCGTCATGACGCCA      660
                ** ******** ******* * **** ***** ********* **** * ****** ***

hNTCP           CTCTTGATTGCCACCTCCTCCCTGATGCCTTTTATTGGCTTTCTGCTGGGTTATGTTCTC      720
pNTCP           CACTTGGTGGCCACCTCCTCCCTTATGCCTTTCACCGGCTTCCTGCTAGGCTACCTTCTT      720
                * **** * ************** ******** *  ***** ***** ** **  ****

hNTCP           TCTGCTCTCTTCTGCCTCAATGGACGGTGCAGACGCACTGTCAGCATGGAGACTGGATGC      780
pNTCP           TCTGCTCTTTTCCGCCTCAATGCTCGGTGCAGTCGCACTGTCTGCATGGAGACTGGATGC      780
                ******** *** *********  ******** ********* *****************

hNTCP           CAAAATGTCCAACTCTGTTCCACCATCCTCAATGTGGCCTTTCCACCTGAAGTCATTGGA      840
pNTCP           CAAAACGTTCAACTCTGCTCCACCATCCTCAACGTGACCTTCCCCCCTGAAGTCATTGGA      840
                ***** ** ******** ************** *** **** ** ***************

hNTCP           CCACTTTTCTTCTTTCCCCTCCTCTACATGATTTTCCAGCTTGGAGAAGGGCTTCTCCTC      900
pNTCP           CCACTCTTCTTCTTTCCTCTCCTCTACATGCTTTTCCAGCTTGGAGAAGGGCTTCTCTTC      900
                ***** *********** ************ ************************** **
```

```
hNTCP      ATTGCCATATTTTGGTGCTATGAGAAATTCAAGACTCCCAAGGATAAAACAAAAATGATC     960
pNTCP      ATTGCCATCTTTCGGTGCTATGAGAAAACCAAGCTTTCCAAAGATAAAATGAAAACGATC     960
           ******** *** **************  ****  * **** *******  **** ****

hNTCP      TACACAGCTGCCACAACTGAAGAAACAATTCCAGGAGCTCTGGGAAATGGCACCTACAAA     1020
pNTCP      TCCGCAGCTGATTCAACAGAAGAAACCATTCCCACAGCACTGGGAAATGGCACCCACAAA     1020
           * * ******   **** ******** *****   *** *************** *****

hNTCP      GGGGAGGACTGCTCCCCTTGCACAGCCTAG--------- 1050
pNTCP      GGGGAAGAGTGCCCCCCAACACAGCCTAGTGTTGTCTAG 1059
           ***** ** *** ****         *
```

```
#  Percent Identity Matrix - created by Clustal2.1
     1: hNTCP       100.00   84.48
     2: pNTCP        84.48  100.00
```

Total RNA from PPH was isolated and cDNA libraries were generated. Nucleotide sequence comparison of pNTCP sequence with hNTCP was conducted via CLUSTAL O analysis. Concordant nucleotide sequences are marked with (*****).

Supplementary Table 2: Amino acid alignment of hNTCP and pNTCP

```
CLUSTAL O(1.2.4) multiple sequence alignment


hNTCP           MEAHNASAPFNFTLPPNFGKRPTDLALSVILVFMLFFIMLSLGCTMEFSKIKAHLWKPKG      60
pNTCP           MEALNESAPINFTLPHNFGKRPTDLALSVILVFMLLIIMLSLGCTMEFGRIRAHFRKPKG      60
                *** * ***:***** *******************::***********.:*:**: ****

hNTCP           LAIALVAQYGIMPLTAFVLGKVFRLKNIEALAILVCGCSPGGNLSNVFSLAMKGDMNLSI      120
pNTCP           LAIALVAQYGIMPLTAFALGKLFRLNNVEALAILICGCSPGGNLSNIFALAMKGDMNLSI      120
                *****************.***:***:*:******:***********:*:***********

hNTCP           VMTTCSTFCALGMMPLLLYIYSRGIYDGDLKDKVPYKGIVISLVLVLIPCTIGIVLKSKR      180
pNTCP           MMTTCSTFLALGMMPLLLYLYSRGIYDGTLKDKVPYGSIVISLILIPIPCTIGIILNTKR      180
                :******* **********:******** ******* .*****:*: *******:*::**

hNTCP           PQYMRYVIKGGMIIILLCSVAVTVLSAINVGKSIMFAMTPLLIATSSLMPFIGFLLGYVL      240
pNTCP           PQYVRYVIKGGTILLILCAIAVTVLSVLNVGKSILFVMTPHLVATSSLMPFTGFLLGYLL      240
                ***:******* *:::**::******.:******:*.*** *:******** ******:*

hNTCP           SALFCLNGRCRRTVSMETGCQNVQLCSTILNVAFPPEVIGPLFFFPLLYMIFQLGEGLLL      300
pNTCP           SALFRLNARCSRTVCMETGCQNVQLCSTILNVTFPPEVIGPLFFFPLLYMLFQLGEGLLF      300
                **** **.** ***.*****************:*****************:********:

hNTCP           IAIFWCYEKFKTPKDKTKMIYTAATTEETIPGALGNGTYKGEDCSPCTA*---    349
pNTCP           IAIFRCYEKTKLSKDKMKTISAADSTEETIPTALGNGTHKGEECPPTQPSVV*    352
                **** **** *  *** * * :* :****** ******:***:* *

#  Percent Identity Matrix - created by Clustal2.1
     1: hNTCP       100.00   80.23
     2: pNTCP        80.23  100.00
```

From the nucleotide sequence comparison conducted above, aa sequence comparison between hNTCP and pNTCP was performed using CLUSTAL O. Concordant aa are marked with (*), aa conservation between groups of strongly similar properties are marked with (:), and aa conservation between groups of weakly similar properties are marked with (.). The aa of exon 2 are denoted in bold font, aa157-165 are highlighted in yellow, and aa 166 and 167 are highlighted in green.

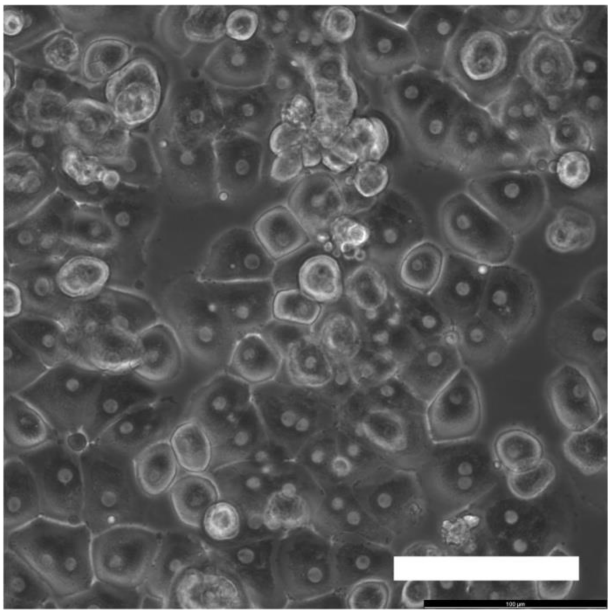

Supplementary Figure 1: Cell morphology of isolated primary pig hepatocytes

PPH were isolated using a 2-step collagenase method. Cells were allowed to attach for 24 hours, and cell morphology and adherence of the PPH were analyzed by phase contrast microscopy (scale bar: 100 µm).

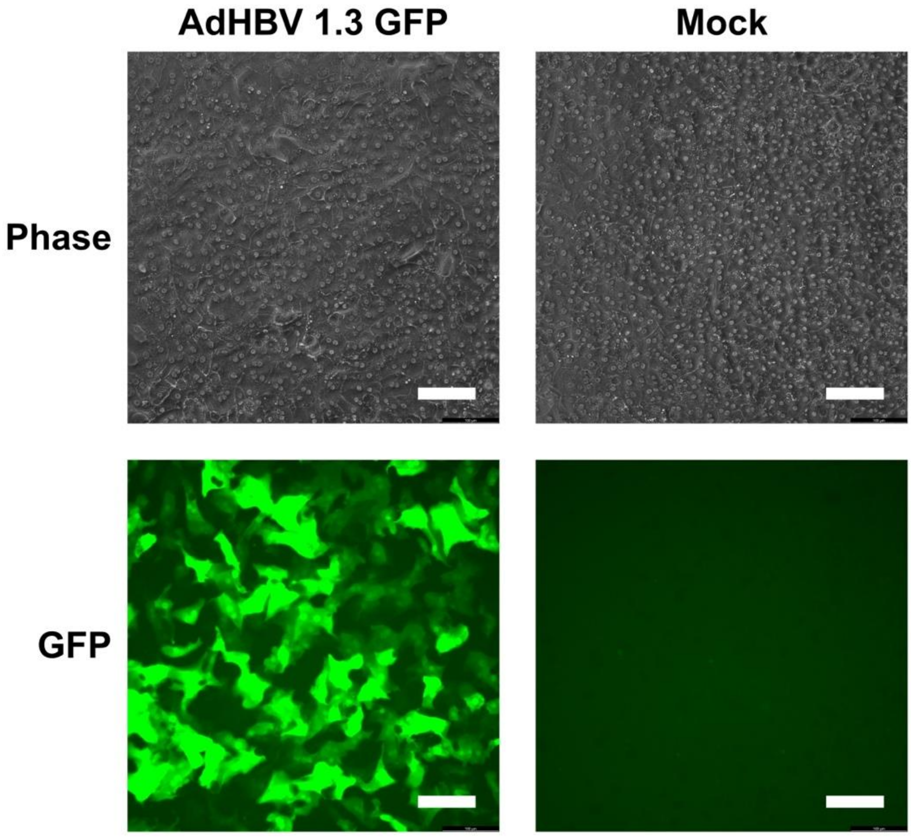

Supplementary Figure 2: Transduction of primary porcine hepatocytes with Ad-HBV1.3
PPH were isolated using a 2-step collagenase method as previously described. Cells were allowed to attach for 24 h, transduced with adenoviral vectors containing a 1.3-fold HBV genome and a GFP expression cassette, and subsequently differentiated in DMSO 2 % for 48 h. Fluorescence microscopy analysis of GFP-positive cells indicates a transduction rate of approximately 75 % of the PPH on day 1 after transduction (scale bar: 100 µm).

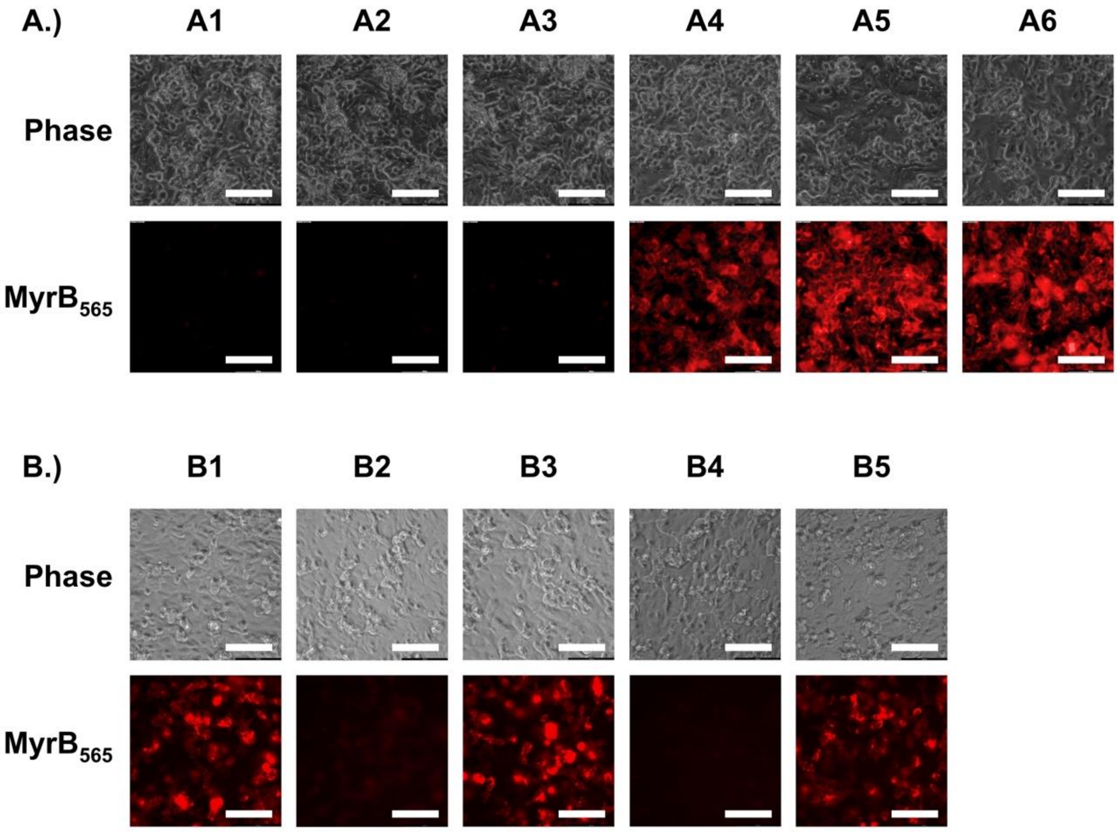

Supplementary Figure 3: Binding of fluorescently labeled Myrcludex B to different NTCP variants
HepG2 cells were transfected with mRNA constructs expressing phNTCP variants encoding for (**A**) block-wise aa exchanges as defined in Fig. 2A or (**B**) single aa substitution as defined in Fig. 2C. Cells were differentiated with 2 % DMSO for 48 h, treated with $MyrB_{565}$ and analyzed via fluorescence microscopy (scale bar: 100 µm). Myrcludex binding was confirmed for the constructs that were identified to allow infection with HBV in Fig. 2B and Fig. 2D.

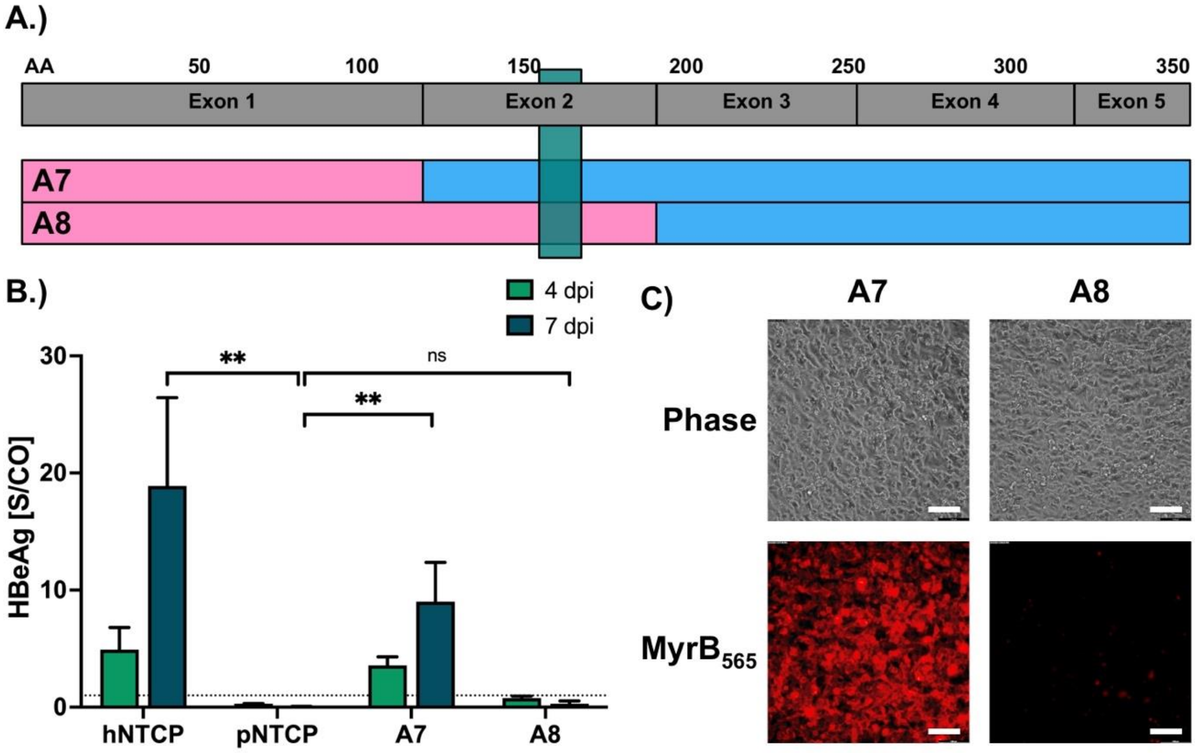

Supplementary Figure 4: Identification of the amino acid domains responsible for blocking HBV infection in pNTCP
(**A**) Overview of the generated phNTCP variants (A7-A8) with a gradual exchange of larger regions of the hNTCP sequence with their porcine counterparts. (**B**) and (**C**) HepG2 cells were transfected with mRNA encoding for these phNTCP variants and differentiated with 2 % DMSO for 48 h. (**B**) Cells were inoculated with HBV (MOI 500 vp/cell). Cell culture supernatants were collected at days 4 and 7 post-infection and analyzed for HBeAg. Experiments were performed in biological triplicates; mean values +/- standard deviation are given. Data were analyzed by one-way ANOVA with Dunnett's correction. ** $p < 0{,}01$, ns = not significant. (**C**) Cells were treated with $MyrB_{565}$ and analyzed via fluorescence microscopy (scale bar: 100 µm).

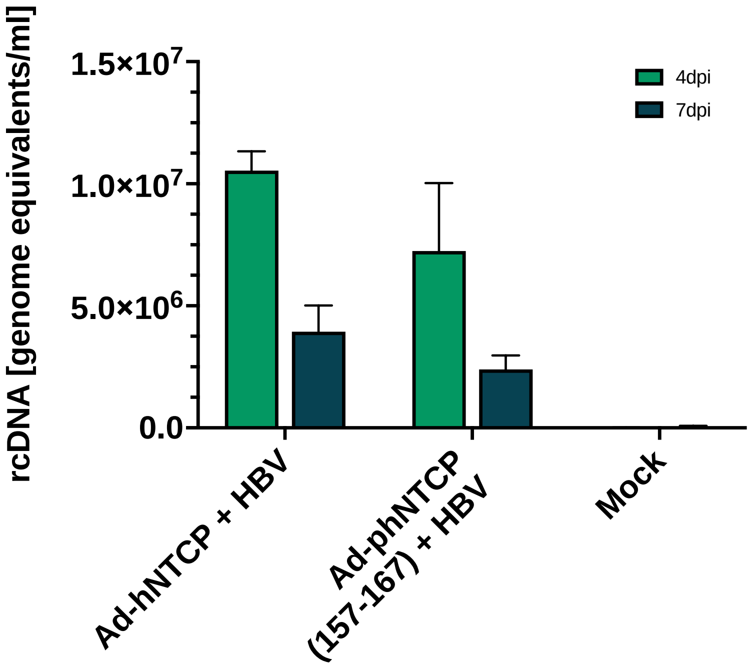

Supplementary Figure 5: Analysis of PPH supernatants for HBV genome equivalents

PPH were isolated using a 2-step collagenase method and seeded on a 6-well plate. Cells were allowed to attach for 24 h, transduced with adenoviral vectors expressing hNTCP or phNTCP (157-167), and subsequently differentiated in DMSO 2 % for 48 h. Cells were then inoculated with HBV (MOI 300 vp/cell) for 24 h. Cell culture supernatants were collected at days 4 and 7 post-infection and analyzed for rcDNA via quantitative PCR. Mean values +/- standard deviation are given.
